# Comparative efficacy and safety of multiple acupoint electrical stimulation therapies for postoperative cognitive dysfunction in cancer patients: a network meta-analysis

**DOI:** 10.1186/s13643-026-03217-7

**Published:** 2026-06-04

**Authors:** Ziyi Chen, Rongze Ma, Hejia Wan, Bingning Zhang, Huiyao Wang, Feifan Wang, Xin Wang, Yuling Zheng

**Affiliations:** 1 Cancer Diagnosis and Treatment Center, The First Affiliated Hospital of Henan University of Chinese Medicine, Zhengzhou, 451200 Henan China; 2 Henan University of Chinese Medicine, Zhengzhou, 450046 Henan China

**Keywords:** Cancer, Postoperative cognitive dysfunction, Electroacupuncture, Transcutaneous electrical acupoint stimulation, Transcutaneous auricular vagus nerve stimulation, Network meta-analysis

## Abstract

**Background:**

Surgery is a primary treatment modality for cancer, yet it is associated with postoperative complications, including postoperative cognitive dysfunction (POCD), which can adversely affect recovery and quality of life. Multiple non-pharmacological approaches, including electroacupuncture (EA), transcutaneous electrical acupoint stimulation (TEAS), and transcutaneous auricular vagus nerve stimulation (taVNS), are employed to mitigate POCD. However, direct comparisons of their relative efficacy are lacking.

**Objective:**

This network meta-analysis aimed to comparatively assess the efficacy of distinct acupoint electrical stimulation modalities—EA, TEAS, and taVNS—in alleviating POCD among surgical oncology patients.

**Methods:**

In accordance with Cochrane guidelines, a systematic review coupled with network meta-analysis was undertaken. Randomized controlled trials (RCTs) investigating acupoint electrical stimulation therapies for POCD in cancer patients were searched in databases including CNKI, Wanfang, VIP, CBM, PubMed, Embase, Web of Science, and Cochrane Library from inception to November 1, 2024. Study quality was assessed using the Cochrane Risk of Bias tool. Network meta-analysis was performed using the BUGSnet package in R 4.3.2. Outcome measures included Mini-Mental State Examination (MMSE) scores and the incidence of POCD on postoperative 1-day, 3-day, and 7-day.

**Results:**

Twenty-three RCTs involving 2055 patients and 6 interventions were included, namely EA, TEAS, taVNS, sham EA, sham TEAS, and blank control. Network meta-analysis revealed that for MMSE scores on postoperative 1-day and 3-day, EA and TEAS showed statistically significant differences compared to blank controls, whereas taVNS did not. By postoperative 7-day, no significant differences were observed among EA, TEAS, taVNS, and blank controls in MMSE scores. For POCD incidence on 1-day and 3-day, EA and TEAS demonstrated significant differences compared to sham EA, sham TEAS, and blank controls. By postoperative 7-day, EA and TEAS remained significantly superior to sham TEAS and blank controls in reducing POCD incidence. Cumulative probability ranking indicated that TEAS had the highest probability of being the best for MMSE scores on 1-day and 3-day, while EA ranked highest for MMSE at 7 days, and for reducing POCD incidence at all time points.

**Conclusion:**

Based on probability rankings, TEAS may be more effective in improving postoperative MMSE scores, whereas EA appears superior in reducing POCD incidence. However, due to modest differences in ranking values and methodological limitations, these findings indicate trends rather than establishing definitive superiority.

**Systematic review registration:**

PROSPERO CRD42024617801.

**Supplementary Information:**

The online version contains supplementary material available at 10.1186/s13643-026-03217-7.

## Simple summary

Cancer surgery can sometimes lead to temporary thinking and memory problems afterward, known as postoperative cognitive dysfunction. Doctors use several non-drug methods involving gentle electrical stimulation at specific points on the body—like electroacupuncture (EA), transcutaneous electrical acupoint stimulation (TEAS), and transcutaneous auricular vagus nerve stimulation (taVNS)—to try to prevent this. However, it has not been clear which method works best. This research compared these three methods by analyzing existing studies. It found that both EA and TEAS improved mental test scores and reduced the risk of cognitive problems more effectively than no treatment, especially in the first few days after surgery. TEAS seemed slightly better for early mental scores, while EA was slightly better at reducing the occurrence of cognitive issues. These findings can help doctors choose the most suitable stimulation method for patients and guide future research to confirm the best approaches for protecting thinking skills after cancer surgery.

**Supplementary Information:**

The online version contains supplementary material available at 10.1186/s13643-026-03217-7.

## Introduction

Surgery is a cornerstone treatment for solid tumors, significantly improving survival rates in eligible patients. However, it is often accompanied by postoperative adverse events and postoperative complications that severely impact patients' quality of life [1–3]. POCD, a prevalent neurological complication in cancer patients, presents with deficits in memory, attention, spatial orientation, verbal fluency, and social cognition [4]. The consequences of POCD may include impaired quality of life due to increased patient dependency, cognitive decline, elevated dementia risk, rising healthcare costs, and increased mortality risk [5]. The etiology of POCD remains unclear but is associated with age, surgical type, inflammatory responses, pain, and anesthesia [6]. Currently, no pharmacological treatments have proven effective for POCD, making early prevention a critical strategy [7].

Acupoint electrical stimulation, a traditional Chinese therapy utilizing low-intensity electrical currents to stimulate specific acupoints, offers advantages such as broad applicability, ease of use, minimal side effects, and cost-effectiveness. It may alleviate POCD through mechanisms including anti-inflammatory effects, antioxidant stress reduction, inhibition of hippocampal neuron apoptosis, and modulation of central cholinergic systems [8–12]. Common clinical modalities include EA, TEAS, and taVNS. While existing research supports the efficacy of certain acupoint stimulation methods in improving postoperative cognitive function and biochemical markers, the optimal intervention remains unclear. Through Bayesian network meta-analysis, we directly compare therapeutic effectiveness against POCD, thereby establishing clinically actionable evidence for practice guidelines.

## Materials and methods

The conduct of this network meta-analysis complied with PRISMA-NMA standards [13], employing a protocol prospectively registered in PROSPERO (CRD42024617801). The PRISMA 2020 checklist is provided in the Supplementary material Table S1.

### Inclusion criteria

P (population): Patients aged ≥ 18 years undergoing tumor resection surgery, regardless of gender or cancer type.

I (intervention): Experimental groups received acupoint electrical stimulation therapies, including EA, TEAS, or taVNS.

C (comparison): Control groups received sham stimulation (sham EA, sham TEAS, sham taVNS) or blank controls.

O (outcome): MMSE scores and the incidence of POCD on postoperative 1-day, 3-day, and 7-day.

S (study design): RCTs published in Chinese or English.

### Exclusion criteria

P (population): studies involving patients with chemotherapy-related cognitive impairment, those undergoing non-oncologic surgery, or patients with comorbid severe psychiatric disorders or baseline cognitive impairment.

I (intervention): studies utilizing adjunctive therapies beyond target interventions (including but not limited to integrated TCM or tuina) were excluded.

C (comparison): non-pharmacological interventions beyond the predefined scope of this review were excluded.

O (outcome): studies without relevant outcome indicators.

S (study design): non-RCTs, such as cohort studies or animal experiments.

### Search methods and strategies

Databases including CNKI, Wanfang, VIP, CBM, PubMed, Embase, Web of Science, and Cochrane Library were systematically searched from inception to November 1, 2024. Supplementary search methods were also employed, including manual searches of the clinical trial registries ClinicalTrials.gov and the Chinese Clinical Trial Registry (ChiCTR) to identify unpublished studies, as well as screening the reference lists of included studies (i.e., snowballing) to further identify relevant literature. The literature search strategy combined controlled MeSH terms with free words, using the appropriate boolean logic operators. The subject words included “acupuncture”, “electroacupuncture”, “transcutaneous electrical acupoint stimulation”, “taVNS”, “tumor”, “cognitive dysfunction”, “POCD”. Table 1 provides the PRISMA-compliant search strategies, illustrated by the PubMed protocol.

**Table 1:** The search strategy of PubMed

Sequence number	Content retrieval
#1	acupuncture [Title/Abstract]
#2	electroacupuncture [Title/Abstract]
#3	EA [Title/Abstract]
#4	transcutaneous electrical acupoint stimulation [Title/Abstract]
#5	TEAS [Title/Abstract]
#6	transcutaneous auricular vagus nerve stimulation [Title/Abstract]
#7	taVNS [Title/Abstract]
#8	#1 OR #2 OR #3 OR #4 OR #5 OR #6 OR #7
#9	tumor [Title/Abstract]
#10	tumour [Title/Abstract]
#11	neoplasm [Title/Abstract]
#12	cancer [Title/Abstract]
#13	malignancy [Title/Abstract]
#14	carcinoma [Title/Abstract]
#15	#9 OR #10 OR #11 OR #12 OR #13 OR #14
#16	cognitive dysfunction [Title/Abstract]
#17	cognitive impairment [Title/Abstract]
#18	cognitive function [Title/Abstract]
#19	neurocognitive disorders [Title/Abstract]
#20	POCD [Title/Abstract]
#21	#16 OR #17 OR #18 OR #19 OR #20
#22	#8 AND #15 AND #21

### Study selection and data extraction

All retrieved records were managed using Zotero software, where duplicates were removed, initial screening was performed through title and abstract review, and full-text reassessment was conducted to finalize study inclusion. A standardized information extraction form was developed. Screening and data extraction were conducted independently by two investigators, and any discrepancies were resolved by consultation with a third investigator.

### Quality assessment

The Cochrane risk-of-bias tool was independently applied by two reviewers to assess study quality, with any disagreements resolved by a third reviewer. Corresponding bias plots were generated using R 4.3.2. Certainty of evidence for the predefined primary outcomes was assessed using the GRADE approach adapted for network meta-analysis via CINeMA, considering within-study bias, across-studies bias, indirectness, imprecision, heterogeneity, and incoherence.

### Statistical analysis

Odds ratios (OR) and 95% credible intervals (CrI) were calculated for dichotomous variables using a binomial likelihood and logit link, whereas mean differences (MD) and 95% CrI were calculated for continuous variables using a normal likelihood and identity link. Statistical significance was defined as a 95% CrI for OR excluding 1 or a 95% CrI for MD excluding 0. The Bayesian network meta-analysis was implemented in R using the BUGSnet package, with all relative effect sizes calculated versus blank control. Following the default BUGSnet specification, independent normal priors were assigned to baseline effects and relative treatment effects, and a Uniform(0, *u*) prior was assigned to the heterogeneity standard deviation (σ) in random-effects models, where u denotes the largest study-specific maximum likelihood estimate.

For model selection, fixed-effect and random-effects models were compared using the Deviance Information Criterion (DIC). A random-effects model was selected when its DIC was at least 5 points lower than that of the fixed-effect model, indicating a meaningfully better model fit. When the DIC difference was less than 5 or the two models were comparable, the fixed-effect model was preferred because it assumes no between-study heterogeneity (τ^2^ = 0) and is more parsimonious. Accordingly, τ^2^ was estimated and reported only for outcomes ultimately analyzed using a random-effects model. The Markov chain parameter configurations were set as follows: 3 Markov chains (n.chain), 1000 adaptation iterations (n.adapt), 20,000 burn-in iterations (n.burnin), and 50,000 posterior iterations (n.iter). Default settings were retained for other parameters. Model convergence was confirmed when the potential scale reduction factor (PSRF) approached 1; otherwise, iterations were increased. Loop inconsistency in evidence networks was evaluated via node-splitting analysis. We generated comparison-adjusted funnel plots to assess publication bias and small-study effects using network effect estimates and examined each pairwise comparison separately. Results were presented as network evidence diagrams, rank probability plots, and heat maps of relative intervention effects. The R code implementing the Bayesian network meta-analysis with the BUGSnet package is provided in the Supplementary material Table S2.

### Transitivity assumption

To assess the transitivity assumption, we qualitatively compared the distribution of prespecified potential effect modifiers across treatment comparisons, including cancer type, type of surgery, age, baseline cognitive status, anesthesia regimen, ASA class, acupoint selection, stimulation duration, and electrical stimulation parameters; details are provided in Table 2.

At the patient level, most studies enrolled older adults with broadly comparable perioperative risk profiles. The distributions of age and ASA class were generally similar across treatment comparisons, although several studies reported incomplete information.

Clinically relevant differences were observed in cancer type and surgical procedure across treatment comparisons, including gastric, colorectal, lung, liver, gallbladder, esophageal, and gynecologic cancers, as well as open and minimally invasive procedures. Because disease type and surgical stress may act as effect modifiers for postoperative cognitive outcomes, these between-comparison differences should be considered when interpreting the network estimates.

There was also variability in intervention characteristics across studies, particularly in acupoint selection, stimulation duration, and electrical stimulation parameters. Although several trials used similar low-frequency dense-disperse wave settings, the exact acupoints and stimulation schedules were not fully uniform.

Substantial variation was observed in intraoperative analgesia and anesthesia regimens across studies, with different combinations of propofol, remifentanil, sufentanil, etomidate, dexmedetomidine, cisatracurium, and related agents. Because perioperative anesthetic management may influence postoperative cognitive outcomes, these differences may represent important effect modifiers and should be considered as a potential source of intransitivity.

The distribution of evidence across the network was unbalanced, with most studies concentrated in TEAS and EA comparisons, whereas taVNS contributed fewer studies. This uneven evidence distribution limited the ability to assess transitivity quantitatively and made the evaluation largely qualitative.

As a result, the plausibility of transitivity was considered acceptable overall, but not guaranteed for every comparison.

## Results

### Literature search

**Fig. 1:**
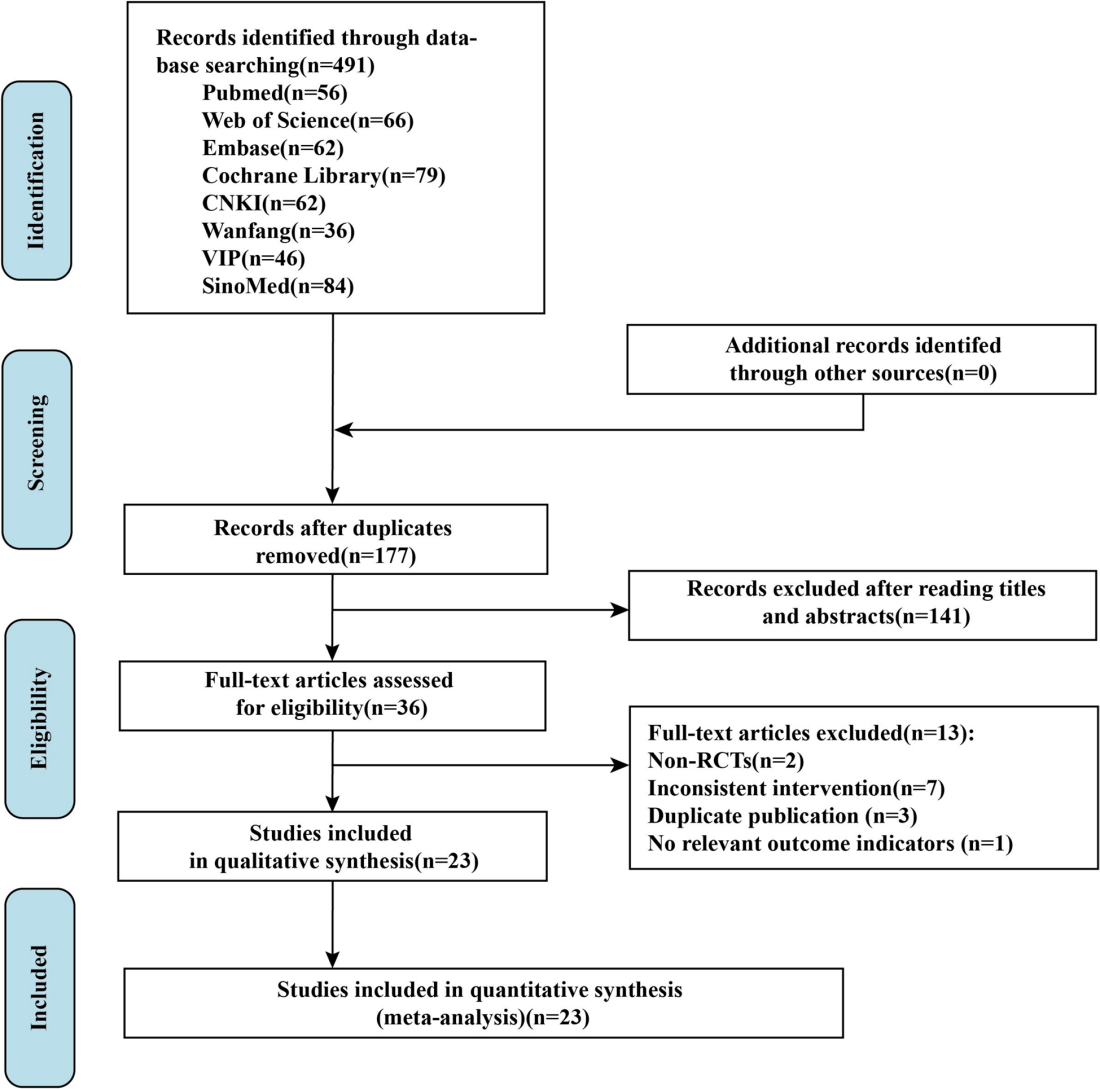
Flow diagram depicting the study selection process

Systematic searches of both Chinese and English databases identified 491 articles. After removing duplicates, initial title/abstract screening, and full-text re-screening, 23 RCTs met the inclusion criteria. The selection flowchart is shown in Fig. 1.

### Study characteristics

The analysis incorporated 23 RCTs [14–36], enrolling a total of 2,055 patients. These comprised 20 two-arm studies [16–35] and 3 three-arm studies [14, 15, 36]. Six interventions were evaluated: TEAS (*n* = 11 studies), EA (*n* = 16), taVNS (*n* = 1), sham TEAS (*n* = 6), sham EA (*n* = 2), and blank control (*n* = 18). Detailed characteristics of the included studies are presented in Table 2.

**Table 2:** Characteristics of included studies

Study	Intervention (E/C)	Sample size (E/C)	Age (E/C)	Gender E/C (male/female)	Type of surgery	Intraoperative anesthesia/analgesia	ASA physical status	Acupoints selectioned	Stimulation protocol	Electrical parameters
Sun HY 2018 [14]	EA vs. Sham EA EA vs. Blank control	20/20 20/20	69.0 ± 2.2/70.3 ± 2.8 69.5 ± 1.7/69.0 ± 2.2	12/8:11/9 12/8:12/8	Radical gastrectomy (for gastric cancer)	Sufentanil + Cisatracurium + Etomidate; Sevoflurane + Sufentanil	I–II	LI4, PC6, ST36, ST37	20 min pre-anesthesia until surgery completion	2/100 Hz Rarefaction-Dense Wave
Zhang D 2022 [15]	taVNS vs.Sham taVNS taVNS vs. Blank control	30/30 30/30	69.3 ± 6.2/69.5 ± 4.5 69.3 ± 6.2/69.8 ± 6.0	17/13:18/12 17/13:16/14	Gastrointestinal tumor surgery	Sufentanil + Dexmedetomidine + Remifentanil	I–II	Auricular vagus nerve area	Pre-op day 1 and post-op days 1, 3, 7 (30 min, twice daily)	10–30 Hz Rarefaction-Dense Wave
Li YF 2023 [16]	TEAS vs. Sham TEAS	48/47	58.12 ± 7.34/56.45 ± 7.26	18/12:20/10	Laparoscopic radical resection for colorectal cancer	Midazolam + Sufentanil + Propofol + Rocuronium Bromide; Propofol + Remifentanil + Sevoflurane	I–II	PC6, LI4, ST36, SP6	30 min pre-anesthesia until surgery completion	2/100 Hz Rarefaction-Dense Wave
Dai M 2024 [17]	TEAS vs. Blank control	50/50	68.72 ± 3.05/67.86 ± 4.43	27/23:25/25	Right hepatectomy	Midazolam + Sufentanil + Cisatracurium + Propofol	I–II	GV20, LI4, ST36, SP6	30 min pre-anesthesia until surgery completion	2–100 Hz Rarefaction-Dense Wave
Ni JW 2015 [18]	TEAS vs. Blank control	30/30	68.7 ± 5.3/69.2 ± 5.0	18/12:20/10	Laparoscopic Dixon procedure for rectal cancer	Propofol + Sufentanil + Remifentanil + Vecuronium Bromide	I–II	GV20, PC6, ST36, SP6	30 min pre-anesthesia until surgery completion	2–100 Hz Rarefaction-Dense Wave
Liu TL 2022 [19]	TEAS vs. Sham TEAS	50/50	70.80 ± 5.41/69.68 ± 4.85	22/28:27/23	Laparoscopic radical colectomy	Fentanyl + Propofol + Cisatracurium; Remifentanil + Propofol	I–II	LI4, PC6, ST36	30 min pre-anesthesia until surgery completion	2–100 Hz Rarefaction-Dense Wave
Tang Y 2017 [20]	TEAS vs. Blank control	45/45	69.6 ± 5.8/70.1 ± 6.3	25/20:24/21	Colorectal cancer surgery	Sufentanil + Etomidate + Rocuronium Bromide; Remifentanil + Propofol	I–III	GV20, GV24	30 min pre-anesthesia until surgery completion	2/100 Hz Rarefaction-Dense Wave
Li XY 2018 [21]	TEAS vs. Blank control	35/32	61.4 ± 9.36/62.7 ± 8.84	18/17:15/17	Thoracoscopic radical resection for lung cancer	Sufentanil + Etomidate + Cisatracurium; Sevoflurane	NA	PC6, ST36	30 min pre-anesthesia until surgery completion	2/100 Hz Rarefaction-Dense Wave
Li XY 2016 [22]	TEAS vs. Blank control	30/30	65.7 ± 6.12/66.5 ± 3.95	18/12:16/14	Thoracoscopic radical resection for lung cancer	Sufentanil + Etomidate + Cisatracurium; Sevoflurane	II–III	PC6, ST36	30 min pre-anesthesia until surgery completion	2/100 Hz Rarefaction-Dense Wave
Tang Y 2020 [23]	TEAS vs. Blank control	45/45	50.17 ± 4.82/49.82 ± 4.19	23/22:20/25	Laparoscopic radical resection for colorectal cancer	Midazolam + Sufentanil + Propofol + Vecuronium Bromide; Sevoflurane + Propofol + Remifentanil	I–II	LI4, PC6, ST36	30 min pre-anesthesia until surgery completion	2/15 Hz Rarefaction-Dense Wave
Liu Z 2017 [24]	EA vs. Blank control	49/49	73.61 ± 11.18/74.26 ± 10.38	25/24:23/26	Tumor resection (gastric cancer, liver cancer, gallbladder cancer)	Midazolam + Penehyclidine + Cisatracurium + Vecuronium Bromide; Propofol + Remifentanil + Fentanyl + Rocuronium Bromide	I–II	GV20, PC6, ST36	30 min pre-anesthesia until surgery completion	2/100 Hz Rarefaction-Dense Wave
Cheng GQ 2020 [25]	EA vs. Blank control	60/60	63.61 ± 11.22/63.57 ± 11.14	40/20:41/19	Gastrointestinal tumor resection	Fentanyl + Midazolam; Propofol	I–II	GV20, PC6, ST36, SP6	20 min pre-anesthesia	Tolerated Rarefaction-Dense Wave
Han XN 2018 [26]	EA vs. Blank control	45/45	68.52 ± 2.44/67.93 ± 2.60	26/19:24/21	Resection of intestinal cancer	Midazolam + Propofol + Fentanyl + Vecuronium Bromide; Propofol + Remifentanil	I–II	GV20, PC6, ST36, SP6	20 min pre-anesthesia until surgery completion	4/20 Hz Rarefaction-Dense Wave
Yu Y 2016 [27]	EA vs. Blank control	49/49	75.6 ± 4.1/75.8 ± 4.0	29/20:31/18	Resection of intestinal cancer	Midazolam + Etomidate + Cisatracurium Besylate + Sufentanil; Propofol + Cisatracurium Besylate	I–II	GV20, PC6, ST36, SP6	NA	NA
Zheng PN 2017 [28]	EA vs. Blank control	56/56	75.82 ± 4.17/74.16 ± 4.21	31/25:31/25	Colorectal cancer resection	NA	NA	GV20, PC6, ST36, SP6	NA	NA
Lin SY 2015	EA vs. Blank control	38/37	69 ± 4/68 ± 3	25/13:3/14	Colorectal cancer resection	Midazolam + Propofol + Fentanyl + Vecuronium Bromide; Propofol + Remifentanil	II–III	GV20, PC6, ST36, SP6	20 min pre-anesthesia until surgery completion	4/20 Hz Rarefaction-Dense Wave
Xie J 2023 [30]	EA vs. Blank control	45/45	67.23 ± 6.23/66.75 ± 5.89	28/17:26/19	Laparoscopic radical resection for rectal cancer	Propofol + Sufentanil + Vecuronium Bromide; Propofol + Remifentanil (Target-Controlled Infusion)	I–II	GV20, PC6, ST36, SP6	30 min pre-anesthesia until surgery completion	2–100 Hz Rarefaction-Dense Wave
Wang QN 2021 [31]	EA vs. Sham EA	40/40	67.5 ± 7.4/67.1 ± 7.0	30/10:28/12	Gynecological tumor resection	Midazolam + Sufentanil + Rocuronium Bromide + Etomidate; Propofol + Remifentanil	II–III	PC6, LI4	10 min pre-anesthesia until surgery completion	2/100 Hz Rarefaction-Dense Wave
Dong XC 2016 [32]	EA vs. Blank control	30/30	71 ± 5/69 ± 4	22/8:24/6	Rectal cancer surgery	Etomidate + Sufentanil + Rocuronium Bromide; Propofol + Remifentanil	I–III	GV20, PC6	30 min pre-anesthesia until surgery completion	2/100 Hz Rarefaction-Dense Wave
Guo F 2023 [33]	TEAS vs. Sham TEAS	55/55	70.09 ± 3.95/70.56 ± 4.59	35/20:28/27	Thoracoscopic pneumonectomy	Midazolam + Sufentanil + Propofol + Rocuronium Bromide; Propofol + Remifentanil (Target-Controlled Infusion)	I–III	GV20, PC6, LI4, ST36	30 min pre-anesthesia until surgery completion	2/100 Hz Rarefaction-Dense Wave
Xi L 2021 [34]	TEAS vs. Sham TEAS	32/32	69.28 ± 7.04/72.06 ± 6.91	22/10:24/8	Radical resection of gastrointestinal tumors	Midazolam + Sufentanil + Cisatracurium; Sevoflurane + Sufentanil + Dexmedetomidine + Cisatracurium + Propofol (BIS 40–60)	II–III	EX-HN3, ST36, PC6	30 min pre-anesthesia until surgery completion,plus 1 day before and 3 days after surgery (30 min daily)	2/100 Hz Rarefaction-Dense Wave
Wang N 2017	EA vs. Blank control	48/48	68.74 ± 5.57/68.36 ± 5.43	28/20:30/18	Subtotal gastrectomy	Midazolam + Fentanyl + Vecuronium Bromide + Propofol; Fentanyl + Propofol	NA	LI4, PC6, ST36, ST37	15–20 min before anesthesia induction	80–90 times/min
Fan W 2022 [36]	EA vs. Sham EA EA vs. Blank control	50/50 50/50	62.66 ± 6.94/63.02 ± 6.72 62.66 ± 6.94/63.64 ± 7.48	26/24:27/23 26/24:28/22	Thoracoscopic esophagectomy for esophageal cancer	Midazolam + Sufentanil + Propofol + Cisatracurium Besylate; Remifentanil + Dexmedetomidine + Cisatracurium Besylate + Sevoflurane	I–II	GV20, PC6, ST36	30 min pre-anesthesia until surgery completion	2/100 Hz Rarefaction-Dense Wave

Note: ①Values for age are presented as mean ± standard deviation. Gender is presented as number of male/female patients. In the “Intervention” and “Sample Size” columns, data for studies with multiple control groups are presented on separate lines within the same cell (e.g., Sun HY 2018)

**②**Abbreviations: *E* experimental group, *C* control group, *ASA* American Society of Anesthesiologists Physical Status classification, *LI4* Hegu, *PC6* Neiguan, *ST36* Zusanli, *ST37* Shangjuxu, *SP6* Sanyinjiao, *GV20* Baihui, *GV24* Shenting, *EX-HN3* Yintang

**③**Electrical parameters: frequencies are presented as reported in the original studies. 2/100 Hz denotes a dense-dispersed wave alternating between 2 and 100 Hz. 2–100 Hz indicates a frequency range (or sweep mode) from 2 to 100 Hz

④Missing data: NA indicates that the information was not available or not reported in the original study

### Quality evaluation

Among the 23 included studies, 20 studies [14, 16–27, 29–35] reported specific methods of random allocation. Nineteen studies [14, 16–26, 29–35] used random number tables, and 1 study [27] used a coin toss, with random sequence generation evaluated as a low-risk item. Three studies [15, 28, 36] mentioned randomized controls but did not describe the random allocation method, and random sequence generation was evaluated as unclear risk.

Allocation concealment was used in 1 study [34] with sealed opaque envelopes and was rated as low risk, whereas the remaining studies [14–33, 35, 36] did not describe the method and were judged as unclear risk.

Regarding blinding of participants and personnel, one study [33] reported blinding of subjects and researchers, with implementation of blinding evaluated as low risk. Two studies [15, 34] reported blinding of participants but did not specify whether personnel were blinded; therefore, performance bias in these two studies was rated as unclear risk. The remaining studies [14, 16–32, 35, 36] did not report the methods used for blinding of participants and personnel and were therefore evaluated as having an unclear risk of bias.

Four studies [17, 19, 33, 34] reported blinding of outcome assessors, with implementation of blinding outcome assessment as a low risk. Nineteen studies [14–16, 18, 20–32, 35, 36] did not report blinding and were evaluated as unclear risk.

Three studies [27, 33, 34] reported dropout cases but did not adequately explain the reasons and were therefore rated as high risk for incomplete outcome data. The remaining 20 studies [14–26, 28–32, 35, 36] either reported no dropouts or provided sufficient explanations for any dropouts and were rated as low risk for incomplete outcome data.

The risk of bias for selective reporting was unclear in all studies.

**Fig. 2:**
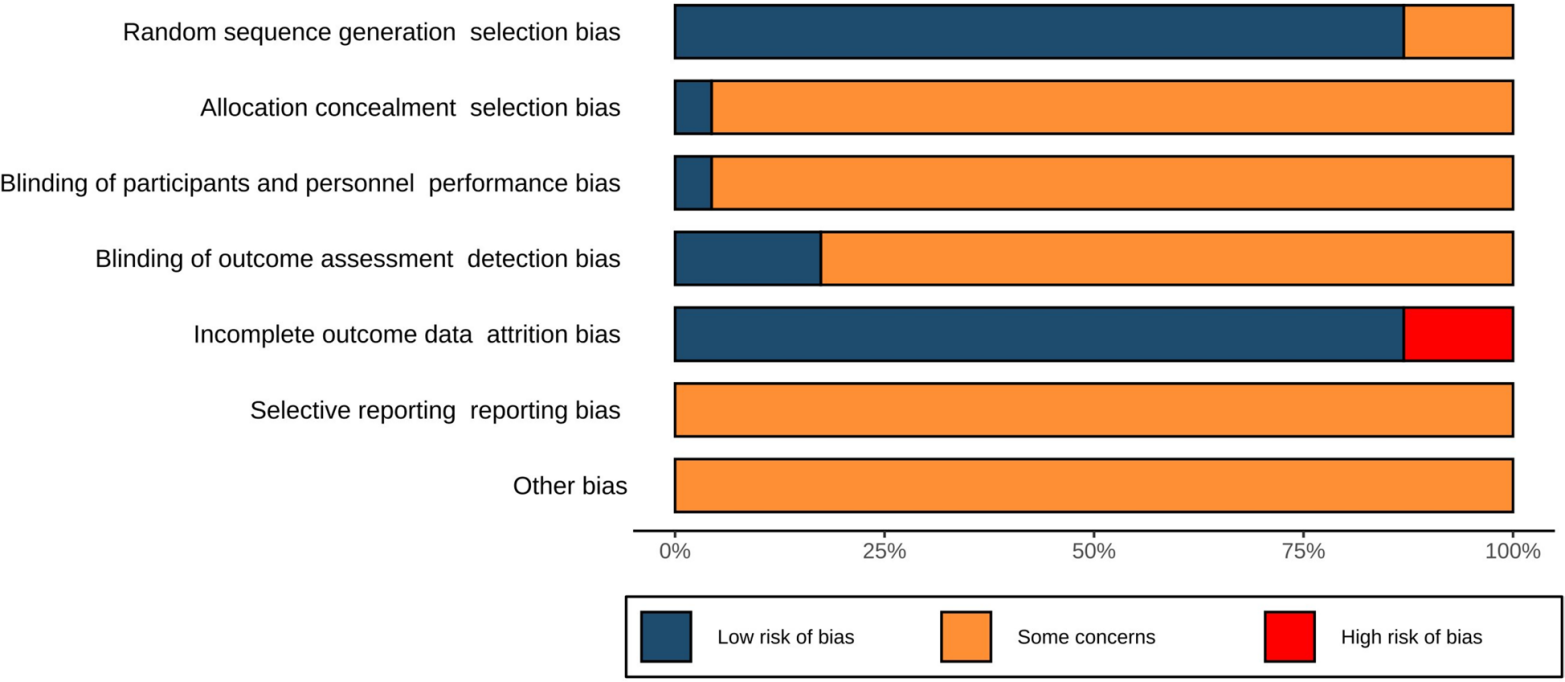
The risk-of-bias evaluation of included studies

The risk of other biases was unclear. The results of the risk of bias evaluation are depicted in Fig. 2.

### Evidence network

**Fig. 3:**
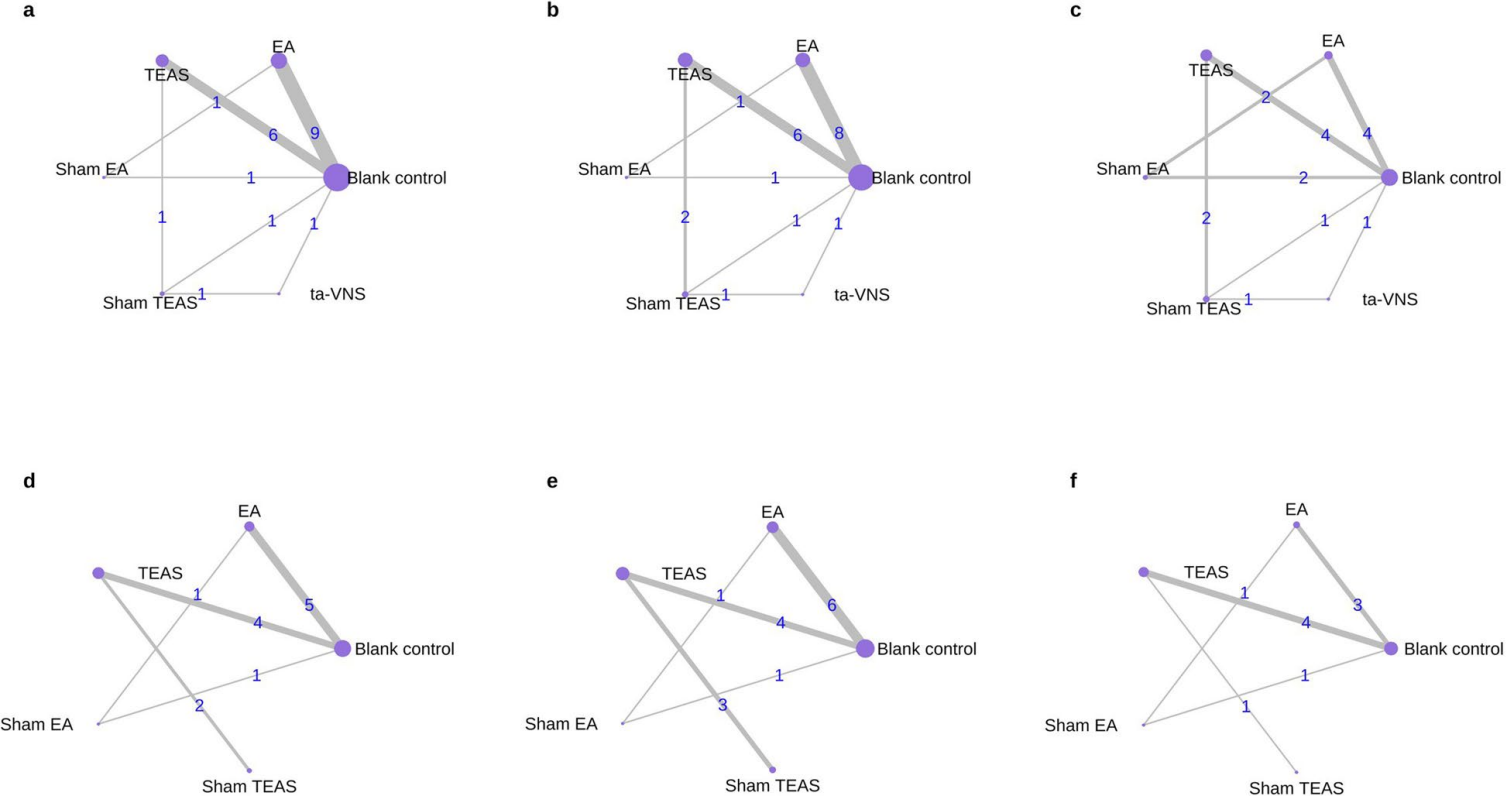
Evidence network diagram. **a** MMSE scores at postoperative 1-day, **b** MMSE scores at postoperative 3-day, **c** MMSE scores at postoperative 7-day, **d** POCD incidence at postoperative 1-day, **e** POCD incidence at postoperative 3-day, **f** POCD incidence at postoperative 7-day

The network of evidence for each outcome is shown in Fig. 3, where nodes represent different interventions, node size corresponds to sample size, lines indicate direct comparisons between interventions, and line thickness reflects the number of studies. Specifically, 17 RCTs reported MMSE scores on postoperative 1-day (totaling 1491 patients); 17 reported MMSE scores on postoperative 3-day (totaling 1384 patients); 11 reported MMSE scores on postoperative 7-day (totaling 980 patients); 11 reported the incidence of POCD on postoperative 1-day (totaling 978 patients); 13 reported the incidence of POCD on postoperative 3-day (totaling 1102 patients); and 8 reported the incidence of POCD on postoperative 7-day (totaling 660 patients). The number of included studies and sample sizes varied across time points, mainly due to (1) some studies only reported outcomes at specific time points; (2) the original data of some studies did not cover all follow-up time points; and (3) this variation primarily stems from actual differences in follow-up schedules in clinical practice, rather than systematic selective reporting. For example, the smaller sample size on postoperative 7-day is mainly because the follow-up protocol of some studies was limited to postoperative 1-day or 3-day.

### Model convergence and consistency test

After 50,000 iterations, the potential scale reduction factors (PSRF) for all parameters were close to 1, indicating satisfactory model convergence. The PSRF results are shown in the Supplementary material Table S3 and Fig. S1.

To choose between fixed-effect and random-effects models, we compared the Deviance Information Criterion (DIC) under both frameworks. A random-effects model was chosen if its DIC was at least 5 points lower than that of the fixed-effect model; otherwise, a fixed-effect model was preferred. For the postoperative 7-day POCD outcome, zero events in individual studies led to unstable DIC estimation, so the posterior mean residual deviance (Dres) was used instead to assess model fit, with the same decision threshold (Dres difference < 5). Accordingly, random-effects models were adopted for the three MMSE outcomes (postoperative 1-, 3-, and 7-day), with posterior mean between-study heterogeneity variance (τ^2^) values of 2.04 (95% CrI = [0.83, 4.61]), 1.56 (95% CrI = [0.61, 3.61]), and 2.34 (95% CrI = [0.69, 6.77]), respectively. Fixed-effect models were selected for POCD incidence at postoperative 1-day and 3-day, as the DIC differences between fixed and random effects were less than 5. For postoperative 7-day POCD incidence, the Dres-based assessment favored a fixed-effect model.

For consistency assessment, outcomes with closed loops were evaluated by comparing the DIC values of the consistency and inconsistency models. No significant inconsistency was identified if the DIC difference was less than 5 or the consistency model had a lower or comparable DIC. For outcomes without closed loops, inconsistency could not be formally assessed; therefore, the consistency model was applied directly. Given the unstable DIC estimation in postoperative 7-day POCD incidence, Dres was also used to confirm model consistency. Overall, no significant inconsistency was detected, and consistency models were ultimately adopted for all outcomes. The DIC and Dres values for model selection and inconsistency assessment are summarized in Table 3.

**Table 3:** Effect model selection of outcome indicator

Outcome indicator	Fixed effects model			Random effects model		
	pD	Dres	DIC	pD	Dres	DIC
MMSE scores at postoperative 1-day	22.1	374.37	396.38	34.75	35.72	70.47
MMSE scores at postoperative 3-day	21.98	199.16	221.14	34.32	35.68	70
MMSE scores at postoperative 7-day	16	139.29	155.29	24.14	24.93	49.07
POCD incidence at postoperative 1-day	15.3	18.84	34.14	16.87	19.2	36.06
POCD incidence at postoperative 3-day	17.29	21.95	39.24	18.78	22.46	41.24
POCD incidence at postoperative 7-day	NaN	19.98	NaN	NaN	18.53	NaN

*Note*: *DIC* represents Deviance Information Criterion, *pD *represents effective number of parameters, *Dres *represents residual deviance. Lower DIC values indicate better model fit. A random-effects model was selected if its DIC was at least 5 points lower than the fixed-effect model; otherwise, the fixed-effect model was preferred. For the outcome of 7-day postoperative POCD, zero events in individual studies caused unstable DIC estimation, so Dres was adopted to evaluate model fit with the same threshold (difference < 5)

### Network meta-analysis

#### Postoperative MMSE scores

##### At postoperative 1-day

There were 17 studies (14–15, 17–18, 20–28, 30, 32–33, 35) reported 1-day postoperative MMSE scores, involving 6 interventions comprising 1491 patients.

**Fig. 4:**
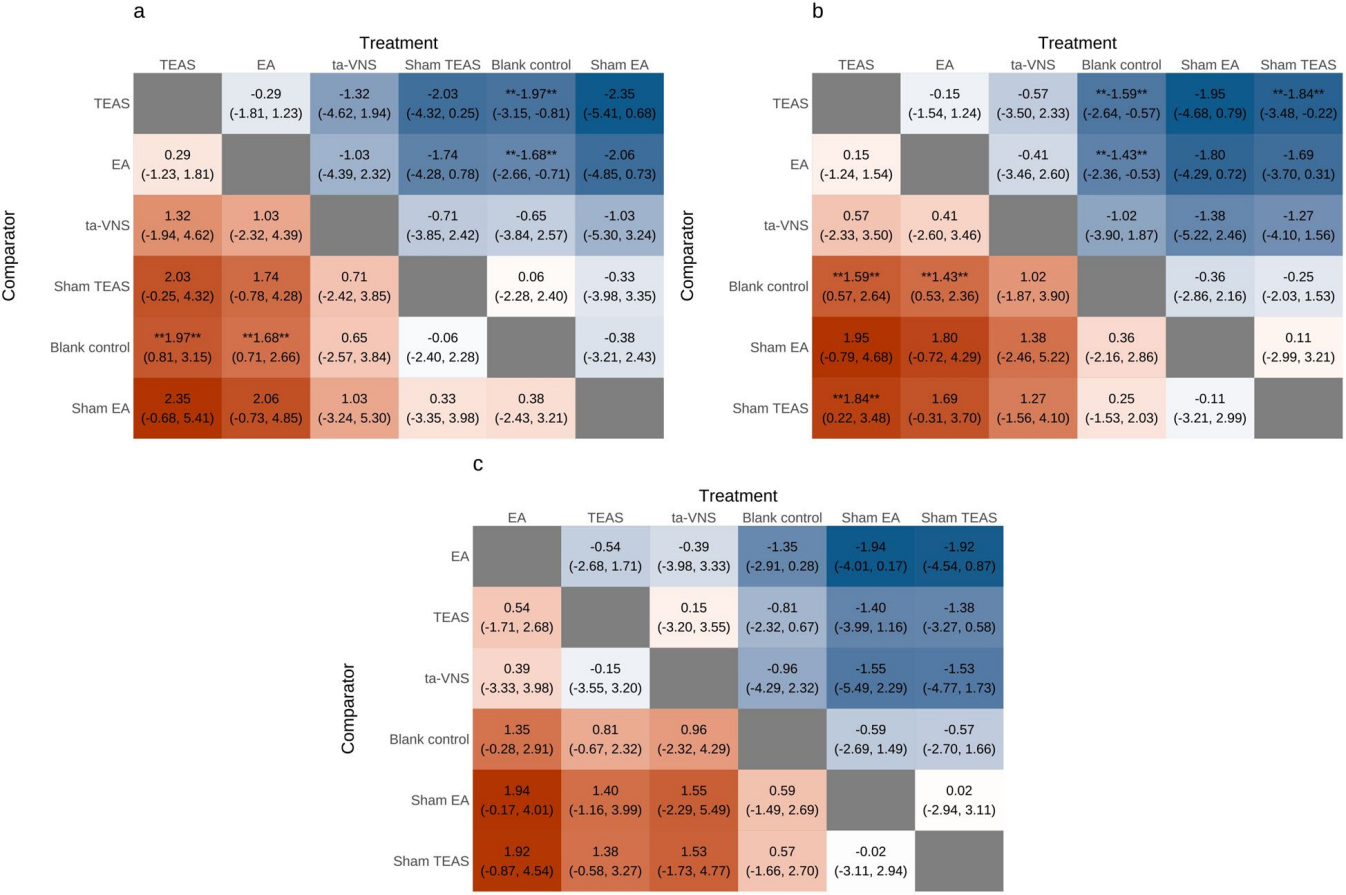
The ranking heat map of MMSE score. **a** 1-day after operation, **b** 3-day after operation, **c** 7-day after operation; ** indicates that the 95% CrI does not include the null value (OR = 1 for dichotomous outcomes; MD = 0 for continuous outcomes)

Network meta-analysis results demonstrated that TEAS and EA significantly improved 1-day postoperative MMSE scores compared to blank control, with statistically significant differences (TEAS: MD = [1.97], 95% CrI = [0.81, 3.15]; EA: MD = [1.68], 95% CrI = [0.71, 2.26]). These results are presented in Fig. 4a.

**Fig. 5:**
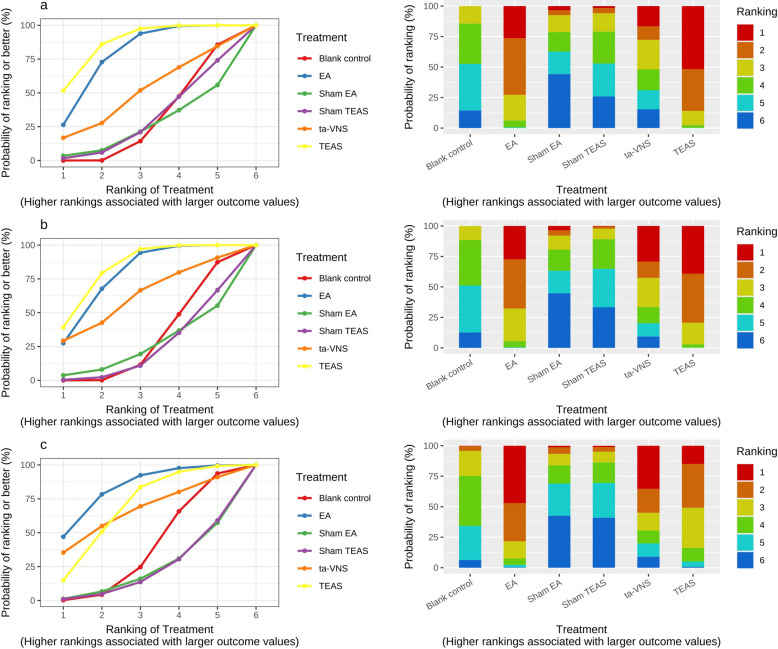
The SUCRA value and rankogram of MMSE score. **a** 1-day after operation, **b** 3-day after operation, **c** 7-day after operation

The SUCRA ranking for interventions improving postoperative 1-day MMSE scores was: TEAS (87.02), EA (78.49), taVNS (49.98), sham TEAS (29.94), blank control (29.48), sham EA (25.08), as shown in Fig. 5a.

##### At postoperative 3-day

There were 17 studies (14–15, 17–18, 20–24, 26–32, 34) reporting 3-day postoperative MMSE scores, involving 6 interventions comprising 1384 patients.

Network meta-analysis results showed that TEAS and EA significantly improved 3-day postoperative MMSE scores compared to blank control (TEAS: MD = [1.59], 95% CrI = [0.57, 2.64]; EA: MD = [1.43], 95% CrI = [0.53, 2.36]); TEAS also outperformed sham TEAS, with statistically significant differences (TEAS: MD = [1.84], 95% CrI = [0.22, 3.48]), as presented in Fig. 4b.

The SUCRA ranking for interventions improving postoperative 3-day MMSE scores was TEAS (83.09), EA (77.81), taVNS (61.80), blank control (29.58), sham EA (24.65), sham TEAS (23.07), as shown in Fig. 5b.

##### At postoperative 7-day

There were 11 studies (14–15, 17–18, 20, 23, 30–33, 36) reporting 7-day postoperative MMSE scores, involving 6 interventions comprising 980 patients.

Network meta-analysis showed no statistically significant differences between interventions, as presented in Fig. 4c.

The SUCRA ranking for interventions improving postoperative 7-day MMSE scores was EA (83.01), TEAS (68.73), taVNS (66.19), blank control (37.75), sham EA (22.48), sham TEAS (21.83), as shown in Fig. 5c.

#### Incidence of postoperative POCD

##### At postoperative 1-day

There were 11 studies (14, 17–20, 23–24, 27–28, 32–33) reporting 1-day postoperative POCD incidence, involving 5 interventions comprising 978 patients.

**Fig. 6:**
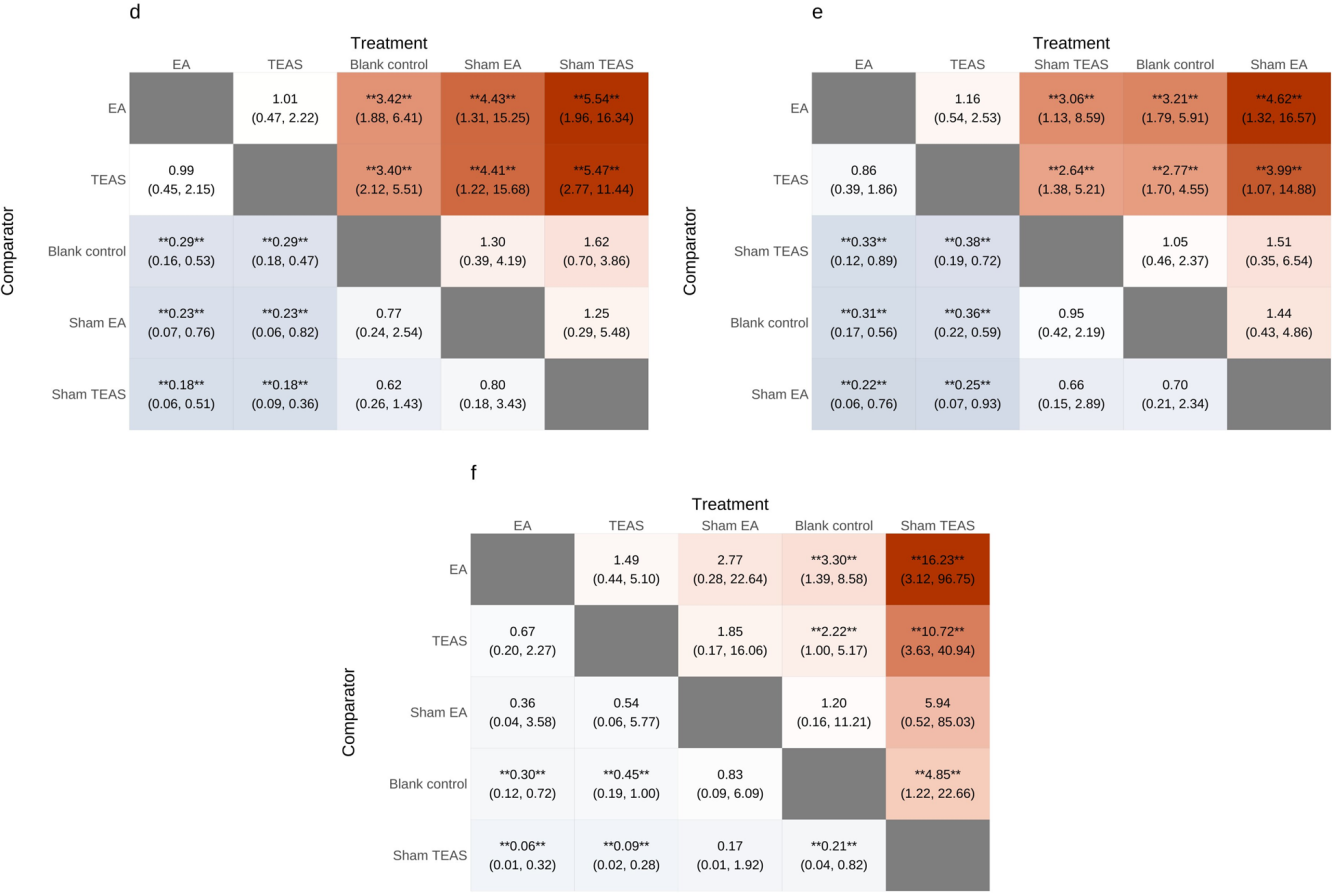
The ranking heat map of the incidence of POCD. **d** 1-day after operation, **e** 3-day after operation, **f** 7-day after operation; ** indicates that the 95% CrI does not include the null value (OR = 1 for dichotomous outcomes; MD = 0 for continuous outcomes)

The network meta-analysis results showed that, compared to sham TEAS, both EA and TEAS significantly reduced the incidence of POCD on postoperative 1-day, with statistically significant differences (EA: OR = [0.18], 95% CrI = [0.06, 0.51]; TEAS: OR = [0.18], 95% CrI = [0.09, 0.36]). Compared to sham EA, both EA and TEAS significantly reduced the incidence of POCD on postoperative 1-day, with statistically significant differences (EA: OR = [0.23], 95% CrI = [0.07, 0.76]; TEAS: OR = [0.23], 95% CrI = [0.06, 0.82]). Compared to blank control, both EA and TEAS significantly reduced the incidence of POCD on postoperative 1-day, with statistically significant differences (EA: OR = [0.29], 95% CrI = [0.16, 0.53]; TEAS: OR = [0.29], 95% CrI = [0.18, 0.47]). The results are presented in Fig. 6d.

**Fig. 7:**
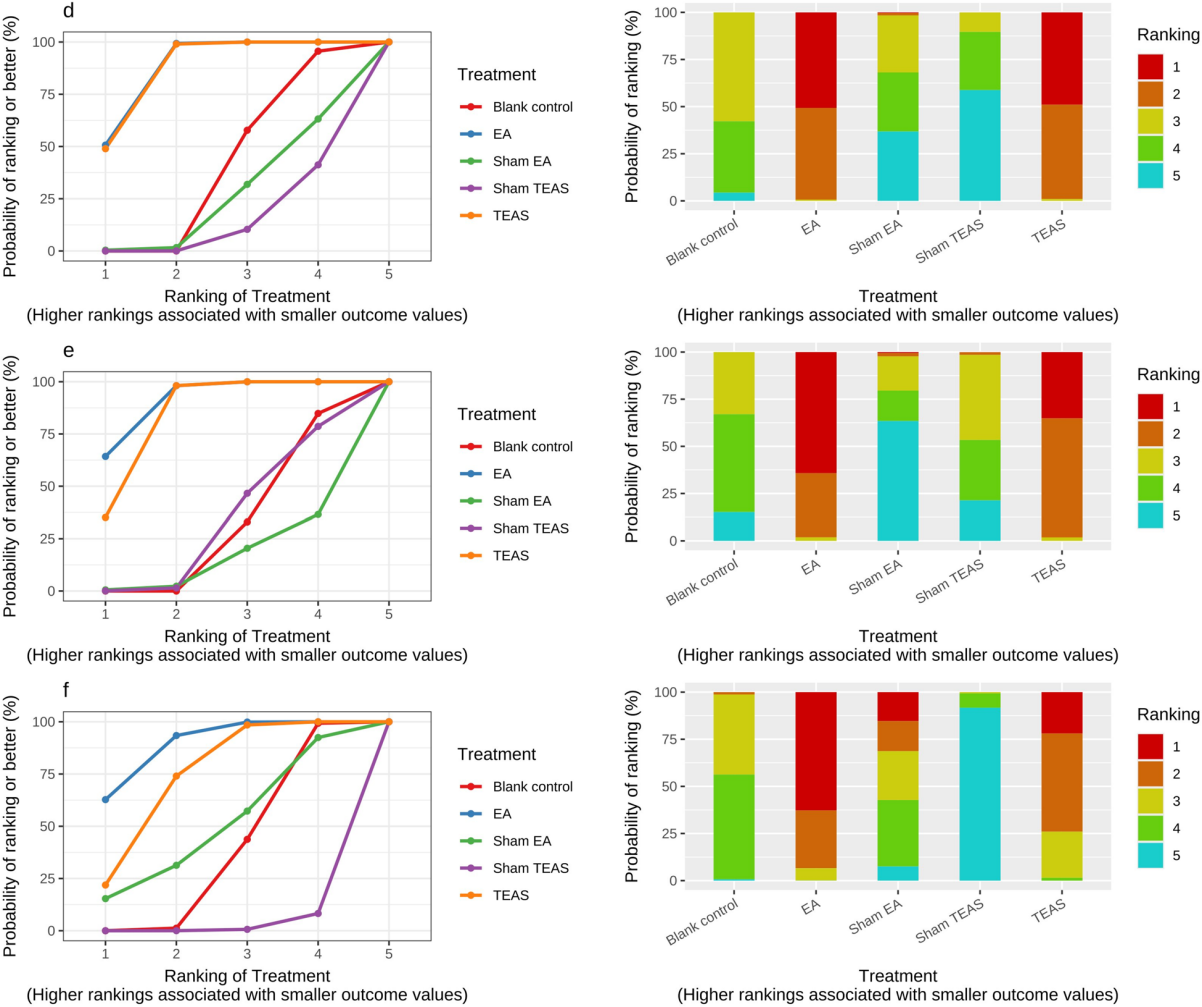
The SUCRA value and rankogram of POCD. **d** 1-day after operation, **e** 3-day after operation, **f** 7-day after operation

The SUCRA ranking for interventions reducing postoperative 1-day POCD incidence was: EA (87.49), TEAS (86.99), blank control (38.35), sham EA (24.26), sham TEAS (12.91), as shown in Fig. 7d.

##### At postoperative 3-day

There were 13 studies [14, 16–20, 23, 24, 27–29, 32, 34] reporting 3-day postoperative POCD incidence, involving 5 interventions comprising 1102 patients.

The network meta-analysis results showed that, compared to sham EA, both EA and TEAS significantly reduced the incidence of POCD on postoperative 3-day, with statistically significant differences (EA: OR = [0.22], 95% CrI = [0.06, 0.76]; TEAS: OR = [0.25], 95% CrI = [0.07, 0.93]). Compared to blank control, both EA and TEAS significantly reduced the incidence of POCD on postoperative 3-day, with statistically significant differences (EA: OR = [0.31], 95% CrI = [0.17, 0.56]; TEAS: OR = [0.36], 95% CrI = [0.22, 0.59]). Compared to sham TEAS, both EA and TEAS significantly reduced the incidence of POCD on postoperative 3-day, with statistically significant differences (EA: OR = [0.33], 95% CrI = [0.12, 0.89]; TEAS: OR = [0.38], 95% CrI = [0.19, 0.72]). The results are presented in Fig. 6e.

The SUCRA ranking for interventions reducing postoperative 3-day POCD incidence was EA (90.45), TEAS (83.31), sham TEAS (31.70), blank control (29.45), sham EA (14.95), as shown in Fig. 7e.

##### At postoperative 7-day

There were 8 studies (14, 17–18, 20, 23, 30, 32–33) reporting 7-day postoperative POCD incidence, involving 5 interventions comprising 660 patients.

The network meta-analysis indicated that EA, TEAS, and blank control were all associated with a significantly lower incidence of POCD on postoperative day 7 relative to sham TEAS (EA: OR = [0.06], 95% CrI = [0.01, 0.32]; TEAS: OR = [0.09], 95% CrI = [0.02, 0.28]; blank control: OR = [0.21], 95% CrI = [0.04, 0.82]). Compared to blank control, both EA and TEAS significantly reduced the incidence of POCD on postoperative 7-day, with statistically significant differences (EA: OR = [0.30], 95% CrI = [0.12, 0.72]; TEAS: OR = [0.45], 95% CrI = [0.19, 1.00]). The results are presented in Fig. 6f.

The SUCRA ranking for interventions reducing postoperative 7-day POCD incidence was EA (89.01), TEAS (73.60), sham EA (49.10), blank control (36.06), sham TEAS (2.24), as shown in Fig. 7f.

### Adverse effect

Six studies reported adverse reactions. Of these, one study reported no adverse reactions in either group, and five studies specifically described adverse reaction profiles, as shown in Table 4. Due to the high heterogeneity in the types, definitions, and reporting time points of adverse events across studies, and the fact that only 6 out of 23 studies (26.1%) provided quantifiable adverse event data, effective quantitative synthesis and analysis were not feasible.

**Table 4:** Summary of reported adverse reactions

Included study	Sample size (E/C)	Intervention (E/C)	Adverse reactions (E)	Adverse reactions (C)
Dai M 2024 [17]	50/50	TEAS vs. blank control	Nausea/vomiting: 1 case; bradycardia: 1 case; chills: 1 case	Nausea/vomiting: 6 cases; bradycardia: 2 cases; chills: 2 cases
Cheng GQ 2020 [25]	60/60	EA vs. blank control	Nausea/vomiting: 1 case	Nausea/vomiting: 6 cases; intestinal obstruction: 2 cases; intestinal adhesion: 1 case
Wang QN 2021 [31]	40/40	TEAS vs. sham TEAS	Pulmonary complications: 9 cases; respiratory depression: 2 cases; bradycardia: 3 cases; tachycardia: 3 cases; hypertension: 4 cases; hypotension: 5 cases; nausea/vomiting: 4 cases	Pulmonary complications: 9 cases; respiratory depression: 3 cases; bradycardia: 2 cases; tachycardia: 2 cases; hypertension: 2 cases; hypotension: 2 cases; nausea/vomiting: 2 cases
Guo F 2023 [33]	55/55	TEAS vs. sham TEAS	Headache: 1 case; nausea/vomiting: 2 cases; local allergic dermatitis: 1 case; discomfort from electrical stimulation: 2 cases	Anorexia: 2 cases; lower limb edema: 1 case; nausea/vomiting: 5 cases; local allergic dermatitis: 1 case
Wang N 2017	48/48	EA vs. blank control	Nausea/vomiting: 3 cases; hypoxemia: 1 case; postoperative agitation: 0 cases; delayed awakening: 2 cases	Nausea/vomiting: 4 cases; hypoxemia: 2 cases; postoperative agitation: 7 cases; delayed awakening: 3 cases

*Note*: *E* experimental group, *C* control group

### Publication bias

**Fig. 8:**
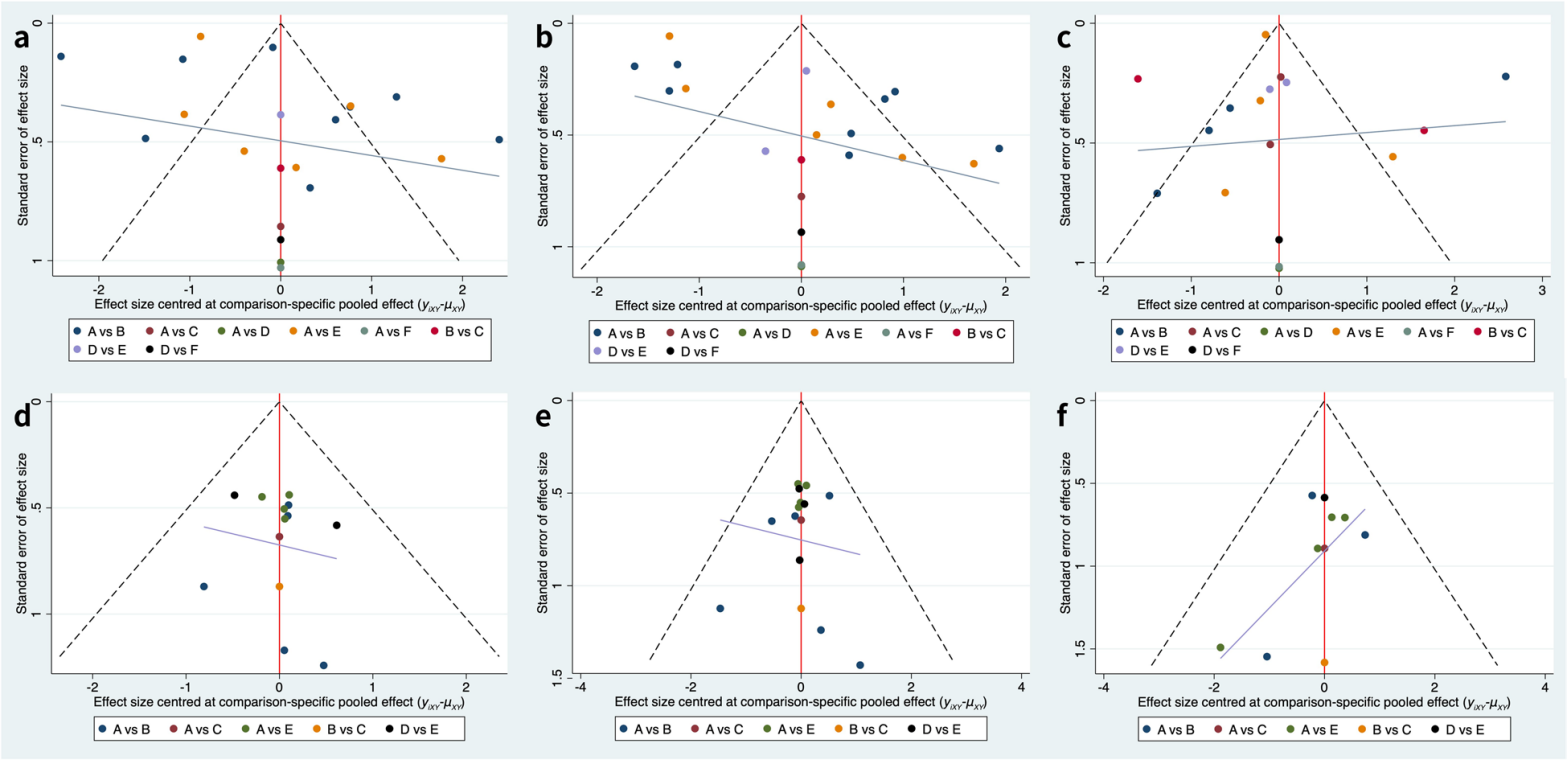
Comparison-corrected funnel plot of each outcome indicator. **a** MMSE scores at postoperative 1-day, **b** MMSE scores at postoperative 3-day, **c** MMSE scores at postoperative 7-day, **d** POCD incidence at postoperative 1-day, **e** POCD incidence at postoperative 3-day, **f** POCD incidence at postoperative 7-day

Comparison-corrected funnel plots were generated for each outcome, as shown in Fig. 8. The funnel plots for postoperative 1-day MMSE scores, postoperative 3-day MMSE scores, and postoperative 7-day MMSE scores were approximately symmetrical; however, some studies fell outside the 95% CrI, suggesting potential small-study effects. The funnel plots for POCD incidence at postoperative 1-day and 3-day displayed near symmetry, with all studies falling within the 95% confidence limits, suggesting a negligible risk of publication bias. The funnel plot for 7-day postoperative POCD incidence showed left-skewed asymmetry, suggesting possible publication bias.

### Certainty of evidence

Using the CINeMA framework, the certainty of evidence was rated according to the prespecified network comparisons. For MMSE, evidence was low to very low across most comparisons. At postoperative 1-day, TEAS and EA improved MMSE scores versus blank control (TEAS: MD = [1.97], 95% CrI = [0.81, 3.15]; EA: MD = [1.68], 95% CrI = [0.71, 2.26]), but the certainty was very low for EA versus blank control and low for TEAS versus blank control. taVNS versus blank control was very low-certainty evidence. At postoperative 3-day, EA and TEAS again improved MMSE scores versus blank control (MD 1.43, 95% CrI 0.53 to 2.36; MD 1.59, 95% CrI 0.57 to 2.64), with low certainty for TEAS and very low certainty for EA. By postoperative 7-day, no statistically significant differences were observed among EA, TEAS, taVNS, and blank control, and the certainty of evidence remained very low. For POCD incidence, EA and TEAS reduced the risk versus blank control at postoperative 1-day (EA: OR = [0.29], 95% CrI = [0.16, 0.53]; TEAS: OR = [0.29], 95% CrI = [0.18, 0.47]) and postoperative 3-day (EA: OR = [0.31], 95% CrI = [0.17, 0.56]; TEAS: OR = [0.36], 95% CrI = [0.22, 0.59]), with moderate certainty for both comparisons at both time points. At postoperative 7-day, EA and TEAS also remained superior to blank control (EA: OR = [0.30], 95% CrI = [0.12, 0.72]; TEAS: OR = [0.45], 95% CrI = [0.19, 1.00]), but certainty decreased to very low. For the indirect comparison EA versus TEAS, CINeMA consistently rated the certainty as very low across all time points, mainly because of within-study bias, imprecision, and incoherence. The detailed ratings for the certainty of evidence are summarized in Table 5.

**Table 5:** Summary of the certainty of evidence for network meta-analysis results based on the CINeMA framework

Outcome	Comparison	Number of studies	Within-study bias	Reporting bias	Indirectness	Imprecision	Heterogeneity	Incoherence	Confidence rating	Reason(s) for downgrading
MMSE POD1	Blank control:EA	9	Some concerns	Low risk	No concerns	No concerns	Major concerns	Major concerns	Very low	["Within-study bias","Heterogeneity","Incoherence"]
	Blank control:TEAS	6	Some concerns	Low risk	No concerns	No concerns	Some concerns	No concerns	Low	["Within-study bias","Heterogeneity"]
	Blank control:taVNS	1	Major concerns	Low risk	No concerns	Major concerns	No concerns	No concerns	Very low	["Within-study bias","Imprecision"]
	EA:TEAS	0	Some concerns	Low risk	No concerns	Major concerns	No concerns	Major concerns	Very low	["Within-study bias","Imprecision","Incoherence"]
	EA:taVNS	0	Major concerns	Low risk	No concerns	Major concerns	No concerns	Major concerns	Very low	["Within-study bias","Imprecision","Incoherence"]
	TEAS:taVNS	0	Major concerns	Low risk	No concerns	Major concerns	No concerns	Major concerns	Very low	["Within-study bias","Imprecision","Incoherence"]
MMSE POD3	Blank control:EA	8	Some concerns	Low risk	No concerns	No concerns	Major concerns	Major concerns	Very low	["Within-study bias","Heterogeneity","Incoherence"]
	Blank control:TEAS	6	Some concerns	Low risk	No concerns	No concerns	Some concerns	No concerns	Low	["Within-study bias","Heterogeneity"]
	Blank control:taVNS	1	Major concerns	Low risk	No concerns	Major concerns	No concerns	No concerns	Very low	["Within-study bias","Imprecision"]
	EA:TEAS	0	Some concerns	Low risk	No concerns	Major concerns	No concerns	Major concerns	Very low	["Within-study bias","Imprecision","Incoherence"]
	EA:taVNS	0	Major concerns	Low risk	No concerns	Major concerns	No concerns	Major concerns	Very low	["Within-study bias","Imprecision","Incoherence"]
	TEAS:taVNS	0	Major concerns	Low risk	No concerns	Major concerns	No concerns	Major concerns	Very low	["Within-study bias","Imprecision","Incoherence"]
MMSE POD7	Blank control:EA	4	Major concerns	Low risk	No concerns	No concerns	Major concerns	Major concerns	Very low	["Within-study bias","Heterogeneity","Incoherence"]
	Blank control:TEAS	4	Some concerns	Low risk	No concerns	Some concerns	Some concerns	No concerns	Very low	["Within-study bias","Imprecision","Heterogeneity"]
	Blank control:taVNS	1	Major concerns	Low risk	No concerns	Major concerns	No concerns	No concerns	Very low	["Within-study bias","Imprecision"]
	EA:TEAS	0	Some concerns	Low risk	No concerns	Major concerns	No concerns	Major concerns	Very low	["Within-study bias","Imprecision","Incoherence"]
	EA:taVNS	0	Major concerns	Low risk	No concerns	Major concerns	No concerns	Major concerns	Very low	["Within-study bias","Imprecision","Incoherence"]
	TEAS:taVNS	0	Major concerns	Low risk	No concerns	Major concerns	No concerns	Major concerns	Very low	["Within-study bias","Imprecision","Incoherence"]
POCD POD1	Blank control:EA	5	Some concerns	Low risk	No concerns	No concerns	No concerns	No concerns	Moderate	["Within-study bias"]
	Blank control:TEAS	4	Some concerns	Low risk	No concerns	No concerns	No concerns	No concerns	Moderate	["Within-study bias"]
	EA:TEAS	0	Some concerns	Low risk	No concerns	Major concerns	No concerns	No concerns	Very low	["Within-study bias","Imprecision"]
POCD POD3	Blank control:EA	6	Some concerns	Low risk	No concerns	No concerns	No concerns	No concerns	Moderate	["Within-study bias"]
	Blank control:TEAS	4	Some concerns	Low risk	No concerns	No concerns	No concerns	No concerns	Moderate	["Within-study bias"]
	EA:TEAS	0	Some concerns	Low risk	No concerns	Major concerns	No concerns	No concerns	Very low	["Within-study bias","Imprecision"]
POCD POD7	Blank control:EA	3	Some concerns	Low risk	No concerns	No concerns	Major concerns	No concerns	Very low	["Within-study bias","Heterogeneity"]
	Blank control:TEAS	4	Some concerns	Low risk	No concerns	Major concerns	No concerns	No concerns	Very low	["Within-study bias","Imprecision"]
	EA:TEAS	0	Some concerns	Low risk	No concerns	Major concerns	No concerns	No concerns	Very low	["Within-study bias","Imprecision"]

*Note*: ①GRADE framework: the certainty (quality) of evidence for each outcome and comparison was assessed using the GRADE approach for network meta-analysis, which considers: within-study bias (risk of bias), reporting bias, indirectness, imprecision, heterogeneity (inconsistency), and incoherence

②Assessment judgments: “Major concerns” in any domain typically leads to downgrading the certainty of evidence by one or two levels. “Some concerns” may or may not lead to downgrading, depending on the overall assessment

③Interpretation of 'Number of studies = 0': for comparisons where the number of direct head-to-head studies is 0 (e.g., EA:TEAS), the estimate is derived entirely from indirect evidence within the network. The assessment of imprecision and other domains for these comparisons is based on the network estimates and their credible interval

④Certainty ratings: the overall certainty of evidence is rated as High, Moderate, Low, or Very low, reflecting the confidence that the true effect lies close to the estimate of the effect

## Discussion

POCD is classified as “forgetfulness” and “dementia” in Traditional Chinese Medicine (TCM) theory. Its pathogenesis primarily involves “vital qi deficiency”, “phlegm stagnation”, and “blood stasis”, with the disease locus situated in the marrow sea. Tumor pathogenesis is attributed to “healthy qi deficiency” combined with phlegm-blood stasis interactions. Surgical interventions further exacerbate qi-blood depletion, leading to “qi-blood insufficiency”, “impoverishment of the marrow sea”, and ultimately POCD.

Currently, no definitive clinical guidelines for POCD management exist globally. However, the implementation of comprehensive preventive strategies involving close collaboration among anesthesiologists, surgeons, geriatricians, and family caregivers has been proposed [37]. This approach holds great potential for reducing the incidence of POCD, facilitating early recovery, and lowering healthcare costs. Consequently, prevention appears to be the most promising strategy for addressing POCD. TCM, guided by the principle of “preventive treatment of pre-disease states”, has demonstrated clinical efficacy. Nevertheless, the relative effectiveness of different TCM interventions—particularly comparative studies among acupoint electrical stimulation modalities—remains underexplored. To address this gap, this study employs a Bayesian network meta-analysis to evaluate acupoint electrical stimulation therapies, aiming to provide evidence-based recommendations for optimizing clinical protocols.

The analysis demonstrated statistically superior effects of EA and TEAS over blank control in improving MMSE scores at postoperative 1-day and postoperative 3-day. Notably, TEAS additionally outperformed sham TEAS in enhancing 3-day postoperative MMSE scores. Based on SUCRA, TEAS had the highest likelihood of being the most effective intervention for improving MMSE scores at 1-day and 3-day postoperatively, followed by EA. However, the differences in SUCRA values between TEAS and EA were not substantial, indicating considerable uncertainty in this ranking and precluding a definitive conclusion of superiority. However, no significant differences emerged between EA/TEAS/taVNS and blank control for 7-day postoperative MMSE scores. A progressive recovery pattern was observed across all groups, with MMSE scores approaching preoperative baseline levels by postoperative 7-day. This is attributable to the typically self-limiting nature of POCD, which rarely persists long-term, although plausible biological mechanisms impacting cerebral protein deposition do exist.

While taVNS showed non-significant MMSE improvements at all time points, its clinical value remains inconclusive due to limited evidence (only 1 included study). These findings warrant cautious interpretation and highlight the need for rigorous trials evaluating taVNS in POCD management.

The analysis revealed statistically significant reductions in POCD incidence with EA and TEAS versus blank control at all time points (postoperative 1-, 3-, and 7-day). Furthermore, both interventions demonstrated superiority over sham EA and sham TEAS in lowering 1-day and 3-day POCD incidence, with additional significant effects against sham TEAS at 7-day. These findings confirm the therapeutic efficacy of EA/TEAS in the 1-day and 3-day postoperative periods while effectively controlling for placebo effects. SUCRA rankings consistently indicated that EA had the highest probability of being the most effective intervention for reducing POCD incidence at all time points, with TEAS ranking second. This trend suggests EA might be a favorable choice for sustained risk reduction, though the evidence does not establish a statistically significant superiority over TEAS.

## Conclusion

This network meta-analysis provides comparative evidence regarding the efficacy of different acupoint electrical stimulation modalities for POCD in cancer patients. The findings suggested that TEAS had the highest probability of being the most effective intervention for improving early postoperative cognitive performance (as measured by MMSE), whereas EA ranked highest for reducing POCD incidence at all assessed time points. These probabilistic rankings, rather than definitive proofs of superiority, can inform clinical consideration. Both interventions significantly outperformed sham stimulation and blank controls, underscoring their potential as non-pharmacological strategies for perioperative neuroprotection. These results not only expand the evidence base for clinical decision-making but also highlight the promise of acupoint electrical stimulation therapies in optimizing cognitive recovery following cancer surgery. Among the six studies that reported adverse events, the observed events were predominantly mild (e.g., nausea/vomiting, local skin discomfort), and no serious adverse events were reported. However, given that most studies did not systematically report adverse events, the overall safety profiles of these interventions remain unclear. More systematic safety data from future studies are needed.

## Limitations

Several limitations of this study should be acknowledged when interpreting the findings. First, the methodological quality of the included RCTs was generally suboptimal, with insufficient reporting on blinding and allocation concealment. Most studies were small, single-center trials published in Chinese, which may have introduced selection and publication bias. Second, the broad inclusion criteria for the study population, combined with imbalanced distributions of key moderating factors for POCD—such as age, type of cancer surgery, anesthesia method, inflammatory response, and pain levels—significantly increased between-study heterogeneity and outcome inconsistency, potentially affecting the transitivity assumption of the network meta-analysis. Third, there was an imbalance in the number of studies and sample sizes across interventions. For example, only one small-scale RCT (involving 30 patients undergoing taVNS treatment) was included for transcutaneous auricular vagus nerve stimulation, resulting in an insufficient body of evidence. This makes it difficult to adequately verify its effect estimates and transitivity, thereby restricting the reliability of the conclusions. Fourth, the follow-up periods were relatively short, with outcomes largely restricted to the first seven postoperative days, thereby precluding evaluation of the long-term effectiveness of these interventions. Fifth, heterogeneity in stimulation parameters, acupoint selection, and treatment protocols may have influenced the consistency of results. Finally, insufficient reporting of adverse events represents a significant limitation of this study, complicating the comprehensive assessment of safety.

Furthermore, there is one deviation from the registered PROSPERO protocol in this study: the registration only prespecified the incidence of POCD as an outcome measure, while this study additionally included the continuous variable of MMSE scores. The literature search revealed that most included studies concurrently reported MMSE scores, and this measure is a recognized standard tool for clinically assessing postoperative cognitive function, capable of providing richer clinical information. This post-hoc addition of outcome measures carries a potential risk of selective reporting bias, and the interpretation of its results should still be approached with caution. This constitutes an important methodological limitation of the present study. Therefore, future large-scale, multicenter RCTs with standardized intervention protocols and extended follow-up durations are warranted to validate these findings and to establish more robust clinical guidelines.

## Supplementary Information

Supplementary Material 1. Table S1. PRISMA 2020 checklist. Table S2. BUGSnet R code for Network Meta-Analysis. Table S3. The PSRF values for model convergence assessment. Figure S1. The PSRF trace and density plots. 

## Data Availability

No datasets were generated or analyzed during the current study.

## Abbreviations

POCD: Postoperative cognitive dysfunction
EA: Electro-acupuncture
TEAS: Transcutaneous electrical acupoint stimulation
taVNS: Transcutaneous auricular vagus nerve stimulation
MMSE: Mini-Mental State Examination
RCT: Randomized controlled trial
OR: Odds ratio
CrI: Credible intervals
MD: Mean difference
DIC: Deviance Information Criterion
PSRF: Potential scale reduction factor
SUCRA: Surface Under the Cumulative Ranking curve
TCM: Traditional Chinese Medicine

## References

[1] Pallan A, Dedelaite M, Mirajkar N, Newman PA, Plowright J, Ashraf S. Postoperative complications of colorectal cancer. Clin Radiol. 2021;76:896–907. 10.1016/j.crad.2021.06.002. 34281707 10.1016/j.crad.2021.06.002

[2] Xu QL, Li H, Zhu YJ, Xu G. The treatments and postoperative complications of esophageal cancer: a review. J Cardiothorac Surg. 2020;15:163. 10.1186/s13019-020-01202-2. 32631428 10.1186/s13019-020-01202-2 PMC7336460

[3] Al-Hilli Z, Wilkerson A. Breast surgery: management of postoperative complications following operations for breast cancer. Surg Clin North Am. 2021;101:845–63. 10.1016/j.suc.2021.06.014. 34537147 10.1016/j.suc.2021.06.014

[4] Needham MJ, Webb CE, Bryden DC. Postoperative cognitive dysfunction and dementia: what we need to know and do. Br J Anaesth. 2017;119:i115–25. 10.1093/bja/aex354. 29161395 10.1093/bja/aex354

[5] Varpaei HA, Farhadi K, Mohammadi M, Khafaee Pour Khamseh A, Mokhtari T. Postoperative cognitive dysfunction: a concept analysis. Aging Clin Exp Res. 2024;36:133. 10.1007/s40520-024-02779-7. 38902462 10.1007/s40520-024-02779-7 PMC11189971

[6] Zhao Q, Wan H, Pan H, Xu Y. Postoperative cognitive dysfunction-current research progress. Front Behav Neurosci. 2024;18:1328790. 10.3389/fnbeh.2024.1328790. 38357422 10.3389/fnbeh.2024.1328790 PMC10865506

[7] Dilmen OK, Meco BC, Evered LA, Radtke FM. Postoperative neurocognitive disorders: a clinical guide. J Clin Anesth. 2024;92:111320. 10.1016/j.jclinane.2023.111320. 37944401 10.1016/j.jclinane.2023.111320

[8] Sun L, Yong Y, Wei P, Wang Y, Li H, Zhou Y, et al. Electroacupuncture ameliorates postoperative cognitive dysfunction and associated neuroinflammation via NLRP3 signal inhibition in aged mice. CNS Neurosci Ther. 2022;28:390–400. 10.1111/cns.13784. 34951130 10.1111/cns.13784 PMC8841296

[9] Huang T, Hong J, Ling J, Zhu L, Zhao W, Zhang X, et al. IL-12p70 induces neuroprotection via the PI3K-AKT-BCL2 axis to mediate the therapeutic effect of electroacupuncture on postoperative cognitive dysfunction. Adv Biol (Weinh). 2025;9:e2400172. 10.1002/adbi.202400172. 39474976 10.1002/adbi.202400172

[10] Wang W, Chen C, Wang Q, Ma JG, Li YS, Guan Z, et al. Electroacupuncture pretreatment preserves telomerase reverse transcriptase function and alleviates postoperative cognitive dysfunction by suppressing oxidative stress and neuroinflammation in aged mice. CNS Neurosci Ther. 2024;30:e14373. 10.1111/cns.14373. 37501354 10.1111/cns.14373 PMC10848091

[11] Zhu B, Zhou Y, Zhou W, Chen C, Wang J, Xu S, et al. Electroacupuncture modulates gut microbiota in mice: a potential target in postoperative cognitive dysfunction. Anat Rec (Hoboken). 2023;306:3131–43. 10.1002/ar.25065. 36094150 10.1002/ar.25065

[12] Zhang Q, Li Y, Yin C, Yu J, Zhao J, Yan L, et al. Electro-acupuncture pretreatment ameliorates anesthesia and surgery-induced cognitive dysfunction via inhibiting mitochondrial injury and neuroapoptosis in aged rats. Neurochem Res. 2022;47:1751–64. 10.1007/s11064-022-03567-3. 35258777 10.1007/s11064-022-03567-3

[13] Hutton B, Salanti G, Caldwell DM, Chaimani A, Schmid CH, Cameron C, et al. The PRISMA extension statement for reporting of Systematic Reviews* For Peer Review incorporating network meta-analyses of health care interventions: checklist and explanations. Ann Intern Med. 2015;162:777–84. 10.7326/M14-2385. 26030634 10.7326/M14-2385

[14] Sun HY, Ji JF, Qian M. Effect of acupuncture combined with general anesthesia under BIS guidance on early postoperative cognitive function in elderly patients undergoing radical gastrectomy for gastric cancer. J Clin Anesthesiol. 2018;34:599–601. 10.12089/jca.2018.06.019.

[15] Zhang D, Wang J, Yang JQ, Wang J, Liu CL, Peng S. Effect of auricular vagus nerve stimulation on postoperative cognitive function in elderly gastrointestinal tumor patients with diabetes mellitus. Xiandai Zhong Xi Yi Jie He Za Zhi. 2022;31:3448–51. 10.3969/j.issn.1008-8849.2022.24.017.

[16] Li YF, Tong YJ. Effect of transcutaneous electrical acupoint stimulation on early cognitive dysfunction and serum inflammatory factors and gastrointestinal hormone in patients underwent laparoscopic radical resection of colorectal cancer. Hebei Zhongyi. 2023;45:1867–71. 10.3969/j.issn.1002-2619.2023.11.024.

[17] Dai M, Yang ZC, Hu RL, Li QQ, Yi JM, Yu Q. Influence of Transcutaneous electrical acupoint stimulation on early postoperative cognitive dysfunction and serum inflammatory factors in elderly liver cancer patients. Zhongguo Zhong Yi Ji Zheng. 2024;33:85–9. 10.3969/j.issn.1004-745X.2024.01.020.

[18] Ni JW, Jiang LM, Zhou YM, Mo YC, Wu Q, Wang JL. Effect of transcutaneous acupoint electrical stimulation on postoperative cognitive function in elderly patients undergoing laparoscopic resection of rectal cancer. Zhonghua Quan Ke Yi Xue. 2015;18:1390–5. 10.3969/j.issn.1007-9572.2015.12.11.

[19] Liu TL. Effects of transcutaneous electrical acupoint stimulationon postoperative cognitive decline in elderly patients andits mechanism. Hebei: Hebei Medical University (2022).10.27111/d.cnki.ghyku.2022.000286

[20] Tang Y, Qing MJ. Effect of transcutaneous acupoint electrical stimulation on postoperative cognitive function in elderly colorectal cancer patients. Xiandai Yi Xue Yu Jian Kang. 2017;33:3623–5. 10.3969/j.issn.1009-5519.2017.23.032.

[21] Li XY. The effect of transcutaneous electrical acupoint stimulation onpostoperative cognitive function in patients undergoing radical thoracoscopic lung cancer operation. Liaoning: China Medical University; 2018.

[22] Li XY, Wei W, Teng XF, Yang YC, Huang X, Zhu JC. Effect of transcutaneous electrical acupoint stimulation on postoperative cognitive function in patients undergoing radical thoracoscopic lung cancer operation. Zhongguo Zhong Yi Yao Xin Xi Za Zhi. 2016;34:1752–5. 10.13193/j.issn.1673-7717.2016.07.063.

[23] Tang Y, Li YJ, Chen Y, Zhao JX, Wang HW. Effects of transcutaneous acupoint electrical stimulation combined with general anesthesia on inflammatory factors, T cell subsets and cognitive function in patients undergoing laparoscopic radical resection of colorectal cancer. Prog Mod Biomed. 2020;20:4571–5. 10.13241/j.cnki.pmb.2020.23.037.

[24] Liu Z, Teng YJ. Effect of acupuncture combined with general anesthesia on cognitive function and related inflammatory factors in elderly patients with tumor resection. China Med Herald. 2017;14:76–9.

[25] Cheng GQ, Fan FY, Wang ZG, Jiang JF, Feng RH. Acupuncture combined with propofol general anesthesia in perioperative period of elderly patients after gastrointestinal tumor resection. Zhongguo Yao Ye. 2020;29:94–7. 10.3969/j.issn.1006-4931.2020.14.032.

[26] Han XN. Effect of acupuncture combined with anesthesia on postoperative cognitive function in elderly patients undergoing intestinal cancer resection. Heilongjiang Zhong Yi Yao. 2018;47:68–9.

[27] Yu Y, Zhong H, Ying XB, Min CM, Mo B, Wang L. Effect of acupuncture combined with general anesthesia on postoperative cognitive function in elderly patients after intestinal cancer resection. Shaanxi Zhong Yi. 2016;37:1070–1. 10.3969/j.issn.1000-7369.2016.08.062.

[28] Zheng PN. Effect of acupuncture combined anesthesia on postoperative cognitive function in elderly patients with colorectal cancer. Clin Med Lond. 2017;37:11–3. 10.19528/j.issn.1003-3548.2017.11.005.

[29] Lin SY, Yin ZL, Gao J, Wen HM, Zhou LJ. Influences of acupuncture anesthesia on postoperative cognitive dysfunction and S-100β protein level of the elderly patients of colorectal cancer resection. Zhongguo Zhen Jiu. 2013;33:63–6. 10.13703/j.0255-2930.2013.01.024.

[30] Xie J, Guo J. Effects of acupuncture anesthesia on postoperative cognitive function and serum levels of S100Β, MMP-9 and BDNF protein in elderly patients undergoing radical resection of rectal cancer. Kang Zhong Liu Yao Xue. 2023;13:84–8. 10.3969/j.issn.2095-1264.2023.01.13.

[31] Wang QN, Zhang JQ, Cui MZ, Suo XY, Xu J, Zhu YF. Effect of acupuncture-drug balanced anesthesia on early cognitive function and short-term clinical prognosis after gynecological tumor surgery of elderly patients. Guoji Yi Yao Wei Sheng Dao Bao. 2021;27:2655–60. 10.3760/cma.j.issn.1007-1245.2021.17.004.

[32] Dong XC, Yue HH, Gao YQ, Xie S, Guan X. Prevention and cure effect of electroacupuncture before anesthesia on postoperative cognitive dysfunction in elderly patients. Chinese Journal of the Frontiers of Medical Science (Electronic Version). 2016;8:82–5. 10.12037/YXQY.2016.09-10.

[33] Guo F, Han R, Sun L, Zheng L, Wang Y, Yan Y, et al. Effect of transcutaneous electrical acupoint stimulation on postoperative cognitive function in older patients with lung cancer: A randomized, double-blind, placebo-controlled trial. Heliyon. (2023) 24;9(9):e19386 10.1016/j.heliyon.2023.e19386 PMC10558345 37809441

[34] Xi L, Fang F, Yuan H, Wang D. Transcutaneous electrical acupoint stimulation for postoperative cognitive dysfunction in geriatric patients with gastrointestinal tumor: a randomized controlled trial. Trials. 2021;22(1):563. 10.1186/s13063-021-05534-9. 34425851 10.1186/s13063-021-05534-9 PMC8383437

[35] Wang N, Ou Y, Qing W. Combined acupuncture and general anesthesia on immune and cognitive function in elderly patients following subtotal gastrectomy for gastric cancer. Oncol Lett. 2018;15(1):189–94. 29391879 10.3892/ol.2017.7262 PMC5769376

[36] Fan W, Liu HL, Li GM, Zheng CJ, Zheng Y, Cao ZY. The effects of acupoint electric stimulation in inflammatory response of tissue and postoperative neurocognitive function undergoing thoracoscopic surgery for esophageal cancer with one-lung ventilation in old patients. Clin J Med Off. 2022;50:793–7. 10.16680/j.1671-3826.2022.08.06.

[37] Wang W, Wang Y, Wu H, Lei L, Xu S, Shen X, et al. Postoperative cognitive dysfunction: current developments in mechanism and prevention. Med Sci Monit. 2014;20(12):1908–12. 10.12659/MSM.892485. 25306127 10.12659/MSM.892485 PMC4206478

